# Evaluation of the accuracy and clinical practicality of a calculation system for patient positional displacement in carbon ion radiotherapy at five sites

**DOI:** 10.1002/acm2.12261

**Published:** 2018-01-25

**Authors:** Yoshiki Kubota, Hayato Hayashi, Satoshi Abe, Saki Souda, Ryosuke Okada, Takayoshi Ishii, Mutsumi Tashiro, Masami Torikoshi, Tatsuaki Kanai, Tatsuya Ohno, Takashi Nakano

**Affiliations:** ^1^ Gunma University Heavy Ion Medical Center Maebashi Gunma Japan; ^2^ Gunma University Graduate School of Medicine Maebashi Gunma Japan; ^3^ Department of Radiology Gunma University Hospital Maebashi Gunma Japan

**Keywords:** automatic system, carbon ion radiotherapy, initial position dependence, patient positioning, ROI size dependence

## Abstract

**Purpose:**

We developed a system for calculating patient positional displacement between digital radiography images (DRs) and digitally reconstructed radiography images (DRRs) to reduce patient radiation exposure, minimize individual differences between radiological technologists in patient positioning, and decrease positioning time. The accuracy of this system at five sites was evaluated with clinical data from cancer patients. The dependence of calculation accuracy on the size of the region of interest (ROI) and initial position was evaluated for clinical use.

**Methods:**

For a preliminary verification, treatment planning and positioning data from eight setup patterns using a head and neck phantom were evaluated. Following this, data from 50 patients with prostate, lung, head and neck, liver, or pancreatic cancer (*n *=* *10 each) were evaluated. Root mean square errors (RMSEs) between the results calculated by our system and the reference positions were assessed. The reference positions were manually determined by two radiological technologists to best‐matching positions with orthogonal DRs and DRRs in six axial directions. The ROI size dependence was evaluated by comparing RMSEs for three different ROI sizes. Additionally, dependence on initial position parameters was evaluated by comparing RMSEs for four position patterns.

**Results:**

For the phantom study, the average (± standard deviation) translation error was 0.17 ± 0.05, rotation error was 0.17 ± 0.07, and Δ*D* was 0.14 ± 0.05. Using the optimal ROI size for each patient site, all cases of prostate, lung, and head and neck cancer with initial position parameters of 10 mm or under were acceptable in our tolerance. However, only four liver cancer cases and three pancreatic cancer cases were acceptable, because of low‐reproducibility regions in the ROIs.

**Conclusion:**

Our system has clinical practicality for prostate, lung, and head and neck cancer cases. Additionally, our findings suggest ROI size dependence in some cases.

## 1 INTRODUCTION

Because particle beams have characteristics such as a Bragg peak and a steep lateral penumbra, they minimize the damage to surrounding normal tissues and effectively concentrate damage onto the tumor.^1^, ^2^ However, the high‐dose radiation still poses some risk to normal tissues and adverse effects can occur if the irradiation position shifts from the target. Therefore, accurate patient positioning is necessary for irradiation treatment.

For photon therapy, patient positioning is often determined using CT images acquired during treatment planning and cone beam (CB) CT images acquired at the time of treatment.^3^, ^4^, ^5^, ^6^ However, simple x‐ray images are commonly used to determine patient positioning for particle therapy at many facilities. There are also some commercial CBCT solutions for particle therapy. For example, CBCT can be included within IBA equipment, although it is probably difficult to adapt this to prevent collision with the irradiation nozzle in a facility with fixed beam lines. Thus, positioning is based on bony structures using the x‐ray images, with a certain margin added for the uncertainty of interfractional motion of the target to assure that the irradiation dose hits the target.

Our facility provides carbon ion radiotherapy as a treatment option for some cancers.^7^, ^8^ Radiography technologists manually perform patient positioning using orthogonal (vertical and horizontal) radiographic images. Manual positioning requires skill and experience because individual differences in positioning can increase the exposure dose with repeated x‐ray images. Additionally, inexperience can result in longer time necessary for positioning. It takes approximately 10–15 min for patient positioning, with 30–60 s for each single matching. Therefore, a high‐precision and high‐speed automatic positioning system is needed to realize safer treatments and increase treatment throughput.

ExacTrac (BrainLAB) is an automatic patient positioning system used in many photon therapy facilities.^9^, ^10^, ^11^ Although this system achieves fast and highly accurate automatic patient positioning, it is incompatible with particle therapy, which requires visualization of bony structures, because all bony structures in the x‐ray image size of the ExacTrac system cannot be seen. Mori et al. reported an automatic patient positioning system for carbon ion radiotherapy.^12^, ^13^ The accuracy of the system was evaluated for tumors in three sites (pelvis, head and neck (H&N), and lung) and the authors reported the optimal metrics for the calculation. However, the system was not evaluated for use in other sites such as liver and pancreas. Additionally, the study did not mention the optimal region size for the calculation at each site. The positioning error could possibly be reduced by choosing the optimal region size for each target site.

We have developed a high‐precision system for calculating patient positional displacement between digital radiography images (DRs) and digitally reconstructed radiography images (DRRs), to reduce the radiation exposure to patients, minimize individual differences among radiological technologists, and decrease the positioning time for carbon ion radiotherapy. In this study, to clarify the practicality of the system, the accuracy of this system was evaluated relative to our setup tolerance using clinical data from patients with tumors at five sites. Moreover, the dependence of calculation accuracy on the size of the region of interest (ROI) and initial positioning parameters were evaluated for each site. It may be useful to know the initial positional dependence to calculate the limits of our system.

## 2 METHODS

### 2.A Imaging devices

**Figure 1:**
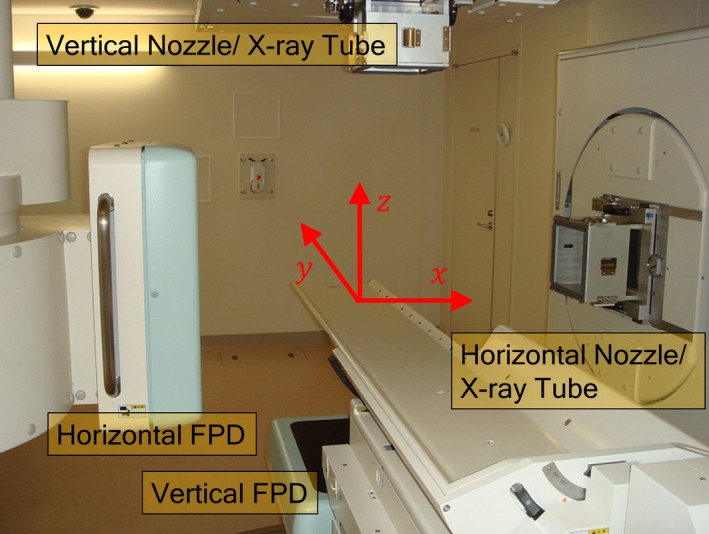
Treatment room at our facility. The x‐ray tubes/flat panel detectors (FPD) and irradiation nozzles are set in the horizontal and vertical directions.

At our facility, CT images are acquired with x‐ray CT (Aquilion LB, Self‐Propelled, Toshiba Medical Systems); treatment planning is performed with the XiO‐N system (Mitsubishi Electric and Eleckta). In the treatment room, horizontal and vertical x‐ray tubes, flat panel detectors (DAR – 8000f, Shimadzu), and carbon beam irradiation nozzles are positioned as shown in Fig. 1. Patient positioning is performed using orthogonal DRs acquired with the flat panel detectors and DRRs reconstructed from CT data during treatment planning.

### 2.B Patient data

Fifty patients treated at our facility for cancer of the prostate, lung, H&N, liver, or pancreas (*n* = 10 each) from April 2010 to November 2015 were randomly selected for retrospective analysis. Each pair of orthogonal images at completion of patient positioning on 1 day during the treatment period was retrospectively analyzed. This study was approved by the Institutional Review Board at our hospital (approval number: 15‐109); all data were anonymized. Orthogonal DRs after patient positioning, treatment planning data, and CT images were evaluated.

### 2.C An algorithm of the system for the calculating patient positional displacement

**Figure 2:**
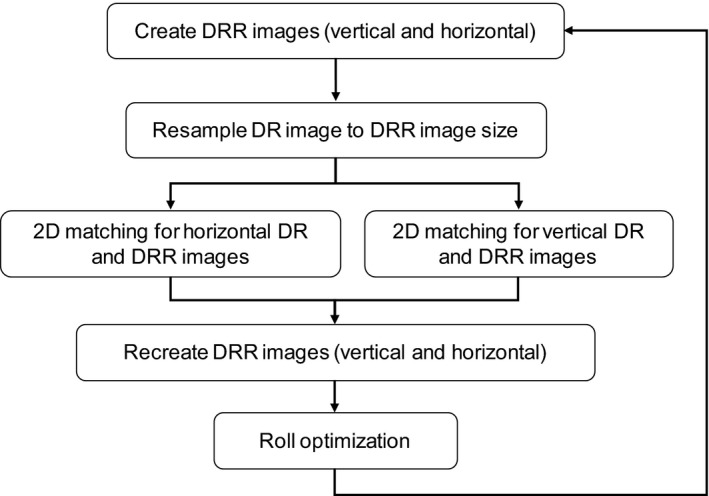
Algorithm flowchart of our calculation system.

The system for calculating patient positional displacement between DRs and DRRs was developed based on a 2D‐3D registration algorithm.^14^, ^15^, ^16^ The system can calculate the positional displacements between the DR and DRR. A flowchart of the calculation algorithm for our system is shown in Fig. 2. The system uses two main procedures, which are 2D matching and roll optimization. The 2D matching step is intended to reduce the calculation costs, as the creation of DRRs, which have a very high calculation cost, only then occurs in the first iteration. The investigation of patient positional displacement between the DR and DRR optimized six parameters $d=(dx,dy,dz,d\theta_{x},d\theta_{y},d\theta_{z})$, indicating lateral, longitudinal, and vertical directions and pitch, roll, and rotation, respectively. The value $d_{0}=\left(dx,dy_{V},d\theta_{z}\right)$ was optimized on the vertical images for 2D matching; $d_{1}=\left(dz,dy_{H},d\theta_{x}\right)$ was optimized on the horizontal images for 2D matching. The variables indicate the lateral axis, vertical axis, and rotation on each image. After 2D matching, dy was calculated as $dy=(dy_{V}+dy_{H})/2$. Additionally, $d_{2}=d\theta_{y}$ was optimized on both images for roll optimization. When $d_{2}$ was calculated in the roll optimization step, the other five parameters $\left(dx,dy,dz,d\theta_{x},d\theta_{z}\right)$, calculated in the 2D matching steps, were directly used. The steepest descent method and the golden section method were used to optimize 2D matching; the golden section method was used for roll optimization.

Zero‐mean normalized cross‐correlation (ZNCC)^1^ 11, 17 was used to assess the similarity between DR and DRR. ZNCC is shown in eq. (1):

(1) $$\mathrm{ZNCC}\left(d_{i}\right)=\frac{\sum_{w}\left(I_{\mathrm{DR}}-\bar{I_{\mathrm{DR}}}\right)\left(I_{\mathrm{DRR}}\left(d_{i}\right)-\bar{I_{\mathrm{DRR}}}\right)}{\sqrt{\sum_{w}{\left(I_{\mathrm{DR}}-\bar{I_{\mathrm{DR}}}\right)}^{2}*\sum_{w}{\left(I_{\mathrm{DRR}}\left(d_{i}\right)-\bar{I_{\mathrm{DRR}}}\right)}^{2}}}\left(i=0,1,2\right)$$

[Correction added on 8^th^ February 2018, after first online publication: Equation was corrected.]

where *W* is the calculation window inside the region of interest (ROI) on vertical or horizontal images, $I_{\mathrm{DR}}$ is the pixel value of each image, and $I_{\mathrm{DRR}}\left(d_{i}\right)$is also the pixel value of each image generated by moving each image or CT volumes with $d_{i}$. The average pixel values in the calculation window for DR and DRR are $\bar{I_{\mathrm{DR}}}$ and $\bar{I_{\mathrm{DRR}}}$, respectively. [Correction added on 8^th^ February 2018, after first online publication: Equation was corrected.] Optimization was performed to minimize an evaluation value calculated as $f\left(d_{i}\right)$, shown in eq. (2). In roll optimization, ZNCC was used for the average of ZNCCs on the vertical and horizontal images. The image size of DR and DRR for all calculation steps used in this study was 256 × 256; the pixel spacing was 0.447 mm. The CT image size was 512 × 512. The pixel spacing in prostate, lung, liver, and pancreas cases was 1.074 mm; the pixel spacing in H&N cases was 0.879 mm. The CT slice thickness at all sites was 2 mm.

(2) $$f\left(d_{i}\right)=1-\mathrm{ZNCC}\left(d_{i}\right)$$

[Correction added on 8^th^ February 2018, after first online publication: Equation was corrected.]

Our calculation system was implemented using a client server system (VT64 Workstation E5‐4S; CPU, Xeon E5‐2670 2.60 GHz (8 cores) × 2; Memory, 32 GB; Operating system, Red Hat Enterprise Linux Server release 6.3: Visual Technology). The calculation program was written in C++ and CUDA 5.0^18^ with open libraries (OpenCV 2.4 and DCMTK 3.6). The client PC (DELL Vostro; CPU Intel Core i7‐3770 3.4 GHz; Memory, 4 GB; Operating system, Windows 7) used a GUI‐based program written in Visual Studio C# with the library OpenCVSharp 2.4.

### 2.D Evaluation method

#### 2.D.1 Calculation of error

To evaluate the accuracy of the system, two radiological technologists with sufficient positioning experience determined the best‐matched position for the bony structures on six parameters between the DR and DRR using the system's manual mode; this position was defined as the reference position. When the reference positions were $x_{\mathrm{ref}},y_{\mathrm{ref}},z_{\mathrm{ref}},\theta_{x,\mathrm{ref}},\theta_{y,\mathrm{ref}},\theta_{z,\mathrm{ref}}$, the error of the system was calculated as the root mean square errors (RMSEs) shown in eqs. (3) and (4). These values were separately calculated in translational and rotational directions.

(3) $$\text{Translation}:\Delta T=\sqrt{({\left(dx-x_{\mathrm{ref}}\right)}^{2}+{\left(dy-y_{\mathrm{ref}}\right)}^{2}+{\left(dz-z_{\mathrm{ref}}\right)}^{2})/3}$$

(4) $$\text{Rotation}:\Delta R=\sqrt{({\left(d\theta_{x}-\theta_{x,\mathrm{ref}}\right)}^{2}+{\left(d\theta_{y}-\theta_{y,\mathrm{ref}}\right)}^{2}+{\left(d\theta_{z}-\theta_{z,\mathrm{ref}}\right)}^{2})/3}$$

Additionally, eq. (5) was used to determine if the errors were within our tolerance. If eq. (5) was satisfied, the calculation result was acceptable.

(5) $$\Delta D=\sqrt{{\left(\frac{\Delta T}{t}\right)}^{2}+{\left(\frac{\Delta R}{r}\right)}^{2}}<1,$$

where t is a translational tolerance factor and r is a rotational tolerance factor. At our facility, setup tolerance is set at 2 mm. 19 Moreover, the angle corresponding to a 2‐mm displacement over 7.5 cm (one half of the maximum irradiation field) is 1.53°. Therefore, t=2 and r=1.53 were used in this study.

#### 2.D.2 Preliminary verification using a head and neck phantom

**Figure 3:**
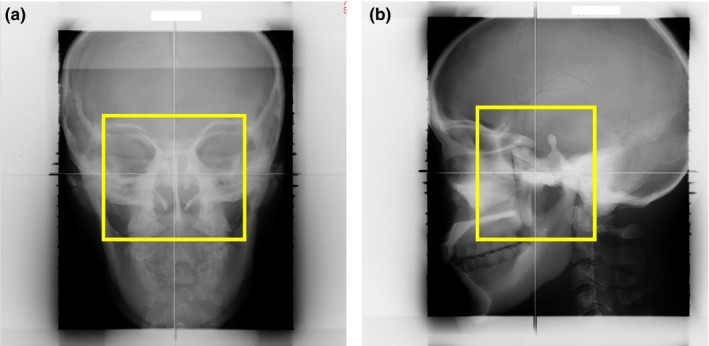
The reference DRs for the head and neck phantom. (a) The horizontal image. (b) The vertical image. The yellow box shows the ROI.

Eight patterns of DR sets and DRRs of a head and neck phantom (Whole Body Phantom PBU‐50, Kyoto Kagaku) were used to verify that our calculation system worked normally. The eight patterns of DR sets are shown in Table 1. The length × width of ROI on the vertical image was 41.2 × 46.5 mm, and these values on the horizontal image were 43.0 × 38.4 mm. The reference DRs are shown in Fig. 3.

**Table 1:** Setup patterns and calculation errors for the head and neck phantom. The values in the total row are the mean and standard deviation for eight patterns

Pattern	Setup						Calculation error								
	x	y	z	$\theta_{x}$	$\theta_{y}$	$\theta_{z}$	x	y	z	$\theta_{x}$	$\theta_{y}$	$\theta_{z}$	ΔT	ΔR	ΔD
1	0.0	0.0	0.0	0.0	0.0	0.0	0.35	−0.07	−0.16	0.16	−0.23	−0.35	0.23	0.26	0.20
2	0.5	0.5	0.5	0.0	0.0	0.0	0.08	−0.12	−0.17	0.17	−0.26	−0.16	0.13	0.20	0.15
3	1.0	1.0	1.0	0.0	0.0	0.0	0.32	−0.20	−0.11	0.16	−0.22	−0.09	0.23	0.16	0.15
4	2.0	2.0	2.0	0.0	0.0	0.0	0.22	−0.25	−0.11	0.19	−0.15	−0.12	0.20	0.15	0.14
5	3.0	3.0	3.0	0.0	0.0	0.0	0.31	−0.06	−0.13	0.14	−0.15	−0.15	0.20	0.14	0.14
6	0.5	0.5	0.5	0.5	0.5	0.5	0.05	−0.01	−0.30	0.18	−0.38	−0.18	0.17	0.26	0.19
7	1.0	1.0	1.0	1.0	1.0	1.0	0.11	0.00	−0.13	0.14	−0.06	−0.08	0.10	0.10	0.08
8	2.0	2.0	2.0	2.0	2.0	2.0	0.18	−0.06	0.09	0.11	0.02	0.00	0.12	0.06	0.07
Total							0.20 ± 0.11	−0.10 ± 0.09	–0.13 ± 0.11	0.16 ± 0.03	−0.18 ± 0.12	−0.14 ± 0.10	0.17 ± 0.05	0.17 ± 0.07	0.14 ± 0.05

#### 2.D.3 Verification of accuracy dependence on ROI size

**Figure 4:**
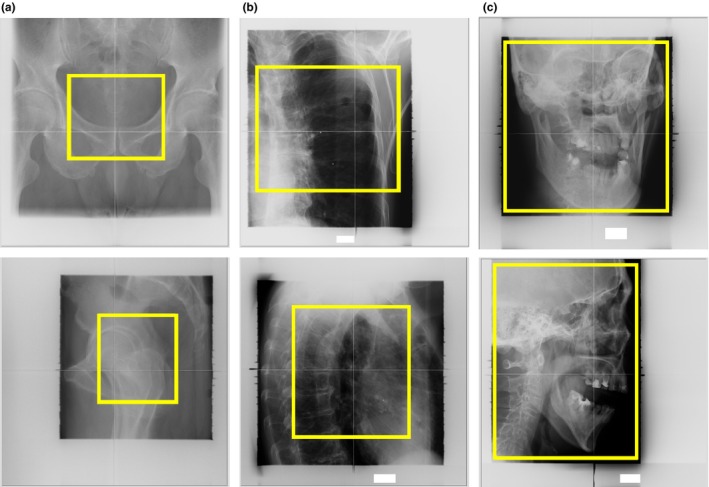
Examples of each ROI size on the DR. (a) Small size for prostate cancer patient. (b) Medium size for lung cancer patient. (c) Large size for H&N cancer patient. The upper row shows the vertical image, the lower row shows the horizontal image, and the yellow box shows the ROI.

To evaluate the accuracy dependence on ROI size at the five sites, three ROI sizes (small, medium, and large) were defined on the horizontal and vertical images. The length × width of small, medium, and large ROI on the vertical image were 41.2 × 46.5 mm, 61.7 × 69.7 mm, and maximum displayed DR, respectively; these values on the horizontal image were 43.0 × 38.4 mm, 64.4 × 57.6 mm, and maximum displayed DR, respectively. The center of both small and medium sizes was set at the isocenter. In most cases, the small size contained the planning target volume, which is an important matching target for the patient positioning, while the medium size usually contained the nearest bones. Examples of the three sizes are shown in Fig. 4. The initial positional values ($x,y,z,\theta_{x},\theta_{y},\theta_{z}$) on the CT images were set to (5 mm, 5 mm, 5 mm, 0.5°, 0.5°, 0.5°) to replicate positioning in clinical use.

#### 2.D.4 Verification of accuracy dependence on initial position parameters

Four patterns of the initial parameters ($x,y,z,\theta_{x},\theta_{y},\theta_{z}$) on the CT images were set: (2 mm, 2 mm, 2 mm, 0.2°, 0.2°, 0.2°), (5 mm, 5 mm, 5 mm, 0.5°, 0.5°, 0.5°), (10 mm, 10 mm, 10 mm, 1°, 1°, 1°), and (20 mm, 20 mm, 20 mm, 2°, 2°, 2°). The accuracy of each pattern was evaluated using the ROI size that had the smallest error, calculated as described in Section 2.D.3, to evaluate the dependence of accuracy on initial position parameters.

#### 2.D.5 Relationship between image correlations and errors

To evaluate the correlation between the calculation result and potential error in each image, averages of vertical and horizontal f(d)and ΔD in the reference position were compared in all cases using the result of the Section 2.D.3 condition, and the correlation coefficient *R* was calculated for each site.

## 3 RESULTS

### 3.A Accuracy of the head and neck phantom

The average (and standard deviation) errors of translation ΔT, rotation ΔR, and ΔD for the head and neck phantom were 0.17 ± 0.05 mm, 0.17 ± 0.07°, and 0.14 ± 0.05, respectively. The calculation results for each pattern are shown in Table 1.

### 3.B Accuracy dependence on ROI size

The calculation results for the five sites are shown in Table 2. The optimal ROI size was small for prostate cancer and liver cancer, large for lung cancer and pancreatic cancer, and medium for H&N cancer. For all sites, the calculation times for small, medium, and large sizes were 25.1 ± 2.91, 24.3 ± 2.3, and 24.1 ± 1.90 s, respectively. At their optimal ROI size, all calculations in the prostate, lung, and H&N cancer patients were acceptable.

**Table 2:** RMSEs and number of acceptable cases using three ROI sizes at five sites. The values in ΔT, ΔR, and ΔD are the mean and standard deviation for 10 patients

ROI	Site				
Size	Prostate	Lung	H&N	Liver	Pancreas
ΔT [mm]					
Small	0.32 ± 0.21	1.43 ± 0.81	0.52 ± 0.24	1.79 ± 1.47	5.85 ± 4.58
Medium	0.41 ± 0.33	0.99 ± 0.57	0.49 ± 0.22	2.38 ± 1.81	4.04 ± 3.30
Large	0.52 ± 0.38	0.99 ± 0.37	0.54 ± 0.26	3.36 ± 3.82	3.22 ± 2.61
ΔR [degree]					
Small	0.37 ± 0.18	0.78 ± 0.39	0.36 ± 0.27	1.22 ± 0.51	3.46 ± 2.00
Medium	0.50 ± 0.50	0.81 ± 0.38	0.28 ± 0.14	1.10 ± 0.51	2.02 ± 1.43
Large	0.43 ± 0.24	0.56 ± 0.30	0.34 ± 0.09	1.95 ± 2.45	1.34 ± 0.92
ΔD					
Small	0.30 ± 0.15	0.90 ± 0.44	0.36 ± 0.20	1.22 ± 0.77	3.87 ± 2.34
Medium	0.41 ± 0.34	0.76 ± 0.29	0.32 ± 0.10	1.44 ± 0.88	2.50 ± 1.77
Large	0.40 ± 0.21	0.63 ± 0.22	0.36 ± 0.11	2.13 ± 2.47	1.88 ± 1.37
Acceptance case					
Small	10	6	10	4	0
Medium	9	8	10	4	2
Large	10	10	10	5	3

### 3.C Accuracy dependence on initial position parameters

**Figure 5:**
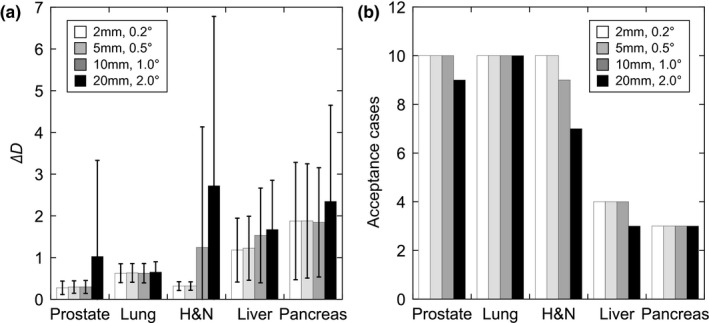
Calculation results and acceptable cases with each initial positional value at five sites. (a) Calculation results. (b) Acceptable cases. Error bars represent standard deviations of the 10 cases for each type of cancer.

Figure 5 shows the calculation results and the number of acceptable cases at each site when the initial positional values were changed. When the initial position parameters for the translation and rotation were 20 mm and 2°, all cases were acceptable only for lung cancer. In contrast, nine prostate cancer cases, seven H&N cancer cases, three liver cancer cases, and three pancreatic cancer cases were acceptable.

### 3.D Relationship between the calculation error and evaluation function

**Figure 6:**
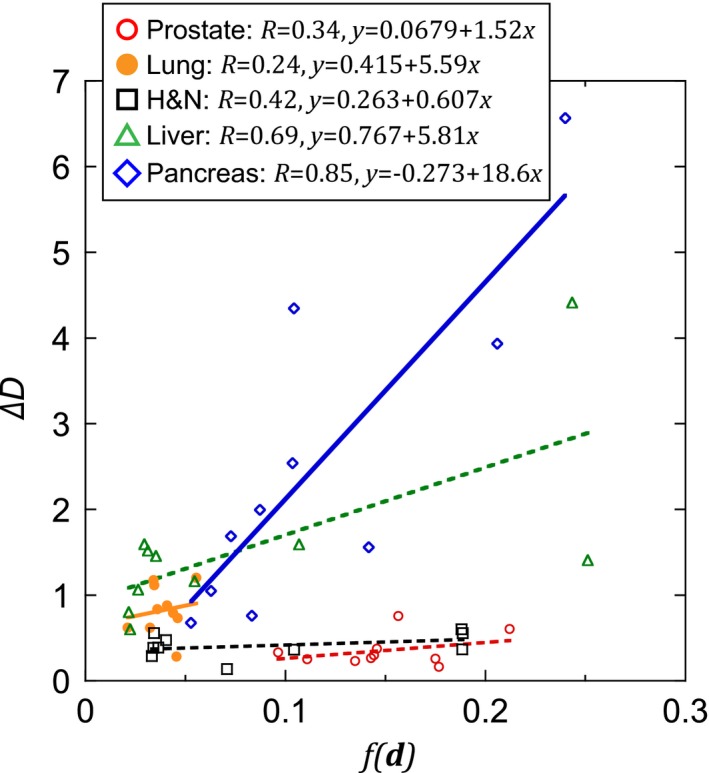
Relationship between evaluation values and calculation errors. Straight lines represent linear approximation for each of the five sites; *R* is the correlation coefficient for each site.

The correlations between the calculation errors for the five sites and the evaluation function are shown in Fig. 6. There was a low correlation for prostate, lung, and H&N (*R* < 0.4); however, a high correlation was found for liver and pancreas (*R* > 0.6).

## 4 DISCUSSION

For the preliminary verification of the head and neck phantom, the average errors were within 0.2 mm and 0.2°, as shown in Table 1. Although these include variations due to the radiological technologists and the error of the calculation system, it was assumed that the system works normally and correctly.

**Figure 7:**
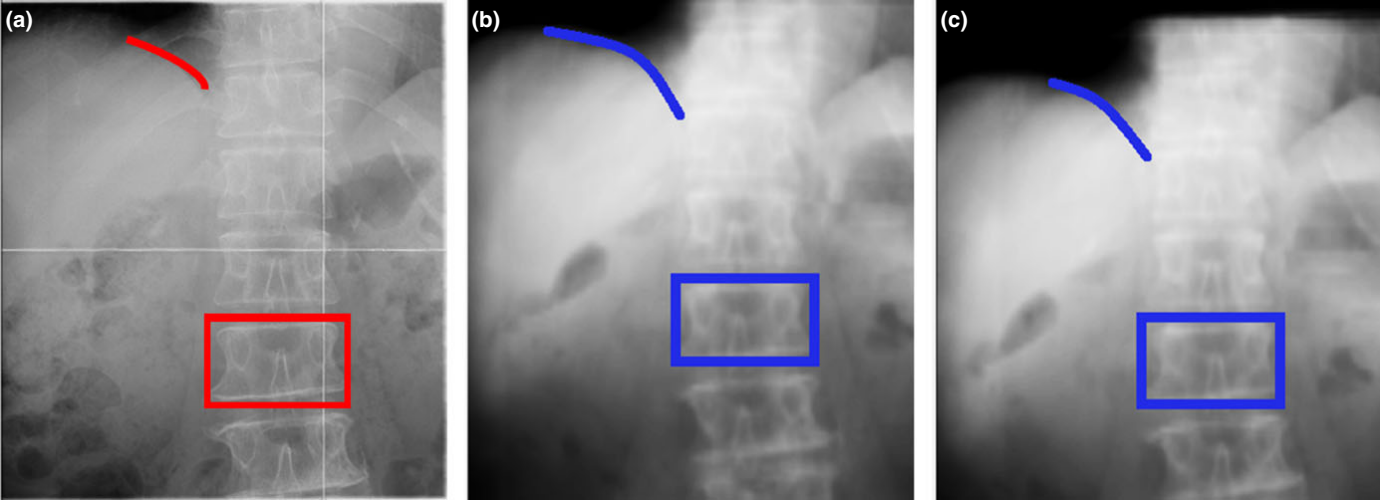
Calculation result images in one case of pancreatic cancer. (a) DR. (b) DRR at the position of calculation result. (c) DRR at reference position. The red box indicates one vertebra on the DR; the red line indicates the diaphragm position. The blue boxes indicate the position of the same vertebra, and the blue lines indicate the position of the diaphragm. The ZNCC for the calculation position shown in (b) is 0.953; the ZNCC for the reference position shown in (c) is 0.757.

Because all cases of prostate, lung, and H&N cancer were acceptable with their optimal ROI sizes (Table 2), they were accurate enough to be feasible at the actual treatment site. However, more than half of liver cancer and pancreatic cancer cases were unacceptable. Here, we consider the cause of large error in one case of pancreatic cancer. The red box in Fig. 7(a) shows the position of one vertebra on the DR; the red line shows the position of the diaphragm. The blue boxes in Figs. 7(b) and 7(c) show the position of the vertebra on the DRR (corresponding to the red box in Fig. 7(a)); the blue lines show the position of the diaphragm (corresponding to the red line in 7 (a)). The positions of the red and blue boxes are almost the same in 7(a) and (c); however, the positions of the red and blue lines are different. Abe et al. reported that the average interfractional error in the marker position was 3.4 mm in the superior–inferior direction.^20^ Additionally, Kawahara et al. reported that the average interfractional error in the diaphragm position was 3.4 mm in the superior–inferior direction.^21^ As shown above, because the positional reproducibility of the liver in the abdomen is low, the patient positioning calculation was misled by the diaphragm position although patient positioning should be performed based on the vertebra position. Considering the correlation values on the images, the ZNCC on the position in Fig. 7(b) is 0.953, and that on the position in Fig. 7(c) is 0.757. The finding that the ZNCC on the calculation was higher than that on the reference position indicates that it is difficult to calculate the optimal patient positioning using the ZNCC alone. In contrast, the images in Figs. 7(a) and 7(c) show that the position of the diaphragm in the DR was different than that in the reference position, whereas the position of the vertebra was almost the same. We assume that the radiological technologist positioned the patient on the basis of the vertebrae, excluding low‐reproducibility regions such as the diaphragm.

Regarding the relationship between evaluation values and calculation errors ΔD shown in Fig. 6, no correlation was found in prostate, lung, or H&N cancer cases, which had relatively small errors. However, in the liver and pancreas cases, which had large errors, a correlation was found. We assume that in cases where the errors were large, the reproducibility of patient conditions (diaphragm position, gas position, and gas volume, for example) between treatment planning and actual treatment was low. Consequently, it is unlikely that the DR and DRR will accurately match on the bony structure for our system, even if the $f(d_{i})$was minimized ($\mathrm{ZNCC}\left(d_{i}\right)$was maximized). Radiological technologists expertly adjust their images on the bony structure by omitting the above‐described low‐reproducibility regions on the basis of their knowledge and experience. It is currently impossible to automatically perform such calculations with our system. To address this problem, it is necessary to add a technique for omitting low‐reproducibility regions from the calculation, as technologists do.

**Figure 8:**
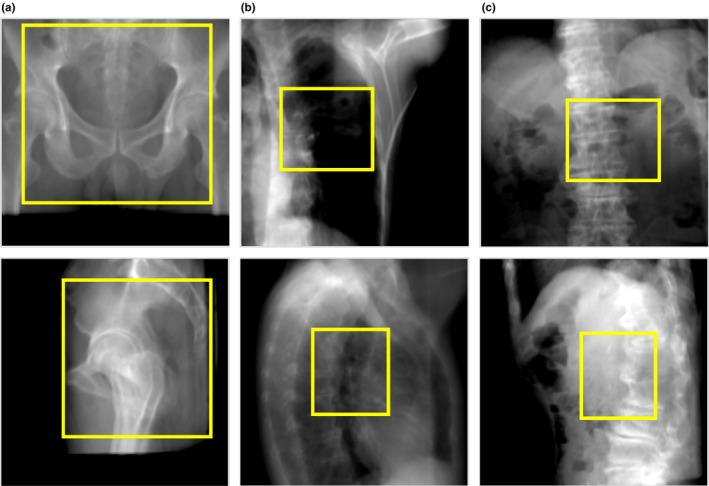
DRR examples of calculation results. (a) Prostate cancer case with large ROI. (b) Lung cancer case with small ROI. (c) Pancreatic cancer case with small ROI. The upper row shows the vertical image and the lower row shows the horizontal image.

To illustrate the dependence of accuracy on ROI size at each site (Table 2), Fig. 8 shows DRR examples of calculation results in a prostate cancer case with a large ROI, a lung cancer case with a small ROI, and a pancreatic cancer case with a small ROI. First, we consider the size dependence of prostate, lung, and H&N cancer cases, which all were acceptable under the optimal ROI size condition. The errors were smallest with the small ROI in the prostate cancer case. This finding can be attributed to the fact that the small ROI did not contain joints such as the hips, whose reproducibility is low. If the center of a small ROI moves the accuracy would be changed; however, the ROI center would not move significantly because the patient positioning is performed using the isocenter (center of the PTV), and therefore the accuracy may be little changed. With a large ROI, as shown in Fig. 8(a), the calculation errors increased because the large ROI often included low‐reproducibility areas such as joints and the bones just below the joints. However, the errors with the small ROI were largest in the case of lung cancer [Fig. 8(b)]. In these cases, the small ROI contained only low‐density regions such as ribs, but not high‐density regions such as vertebrae and shoulder blades. We found that the errors were large if the reproducibility of the shoulder blades was low. In contrast, calculation accuracy was not dependent on ROI size in H&N cancer cases.

In liver cancer cases, the error was smallest with a small ROI. As shown in Fig. 7, if the ROI was large, the diaphragm, whose reproducibility is low, was often included within the ROI. This inclusion caused increased errors because the calculation was affected by different positions of the diaphragm. In contrast, in pancreatic cancer cases, the error was smallest with a large ROI. Kumagai et al. and Houweling et al. reported that the reproducibility of the volume or position of gas in the bowel surrounding the pancreas was very low.^22^, ^23^ Therefore, as shown in Fig. 8(c), when the ROI was small, the relative size of the low‐reproducibility regions within the ROI increased, consequently increasing the error.

In cases of liver and pancreatic cancer with low calculation accuracy, the following two methods can be used to increase calculation accuracy: enlarging the ROI to include the bony structures necessary for calculation or excluding low‐reproducibility regions such as the diaphragm, gas regions, and joints for the calculation.

The average calculation times for each ROI size showed little difference. There are two possible reasons for this difference. One is that the calculation did not converge in cases with large error such as the liver or pancreas. Another is that the calculation system involves extra processing other than the displacement calculation, such as outputting of the log for the verification. As manual matching by the radiological technologist took approximately 30–60 s, the current calculation time (approximately 25 s) may be less than the manual matching time. However, we think this is still too long for introduction to clinical sites, and we expect that the calculation time could be reduced to within 10 s by inclusion of preliminary processing and exclusion of the extra processing.

In this study, the calculation accuracy was evaluated under several initial positional conditions (2 mm, 0.2°; 5 mm, 0.5°; 10 mm, 1°; and 20 mm, 2°). In our previous study, the average of the absolute value of the initial deviation before positioning in prostate cancer cases was 3.0 ± 3.4 mm (maximum value, 14.8 mm).^1^ 17 In the case of patient positioning at our facility, because laser alignment is performed before x‐ray image acquisition, there is no large error. Therefore, from the results in Fig. 5, we assume that the calculation in almost all cases of prostate, lung, and H&N cancer might be acceptable. However, the calculation results for three H&N cases were unacceptable when the initial value was 20 mm and 2°. If the initial positional error is greater than 10 mm, it is necessary to devise a calculation.

The limitations of this study include the low number of cases included. Only 10 cases were analyzed per site. Different cases might have had different results. Thus, further analyses including a greater number of cases are necessary in the future. Additionally, evaluation of special cases, such as patients with metal implants, is necessary.^24^

The verifications performed in this study tested only one image with some errors. To introduce this system to clinical sites, it is necessary to verify its effectiveness in actual positioning with testing involving repeating the calculating errors and moving a couch based on the calculated results.

The calculation system was evaluated on the basis of bony structures. The tumor matching method can improve the target delivered dose for liver or lung cancer cases.^20^, ^25^, ^26^, ^27^ Either the automatic marker or soft tissue alignment method^28^, ^29^ should be included in the calculation system to realize the tumor matching method for patient positioning.

## 5 CONCLUSION

In this study, we developed a system for calculating patient positional displacement between DRs and DRRs, evaluated its practicality for patient data at five lesion sites, and assessed its ROI size dependency at these five sites. We found that almost all prostate, lung, and H&N cancer cases were acceptable for our setup tolerance in clinical practice. Additionally, our comparison of calculation errors for each ROI size suggested the causes of decreased calculation accuracy. In the future, it is necessary to determine how to exclude low‐reproducibility regions from the calculation to improve accuracy in tumors of the liver and pancreas.

## CONFLICTS OF INTEREST

The authors declare no conflict of interest.
